# Hyperplastic polyposis syndrome: endoscopic imaging, phenotypic charcteristics and molecular pathways

**DOI:** 10.1186/1897-4287-10-S2-A19

**Published:** 2012-04-12

**Authors:** KS Boparai, E Dekker, CJM van Noesel

**Affiliations:** 1 Department of Gastroenterology and Hepatology, Academic Medical Center, Amsterdam, The Netherlands; 2 Department of Pathology, Academic Medical Center, Amsterdam, The Netherlands

## Phenotypic characteristics

Hyperplastic polyposis syndrome (HPS) is a heterogeneous condition involving colorectal serrated polyps and is associated with an increased colorectal cancer (CRC) risk. We evaluated the clinicopathological features of HPS patients and their first-degree relatives. Previously published case series report CRC at clinical presentation in up to 50% of HPS patients. We analyzed the risk of developing CRC after HPS diagnosis and which variables are associated with CRC in a large multi-centre cohort of HPS patients undergoing endoscopic surveillance. Furthermore, the prevalence of polyps and CRC in first-degree relatives is described and compared with the background prevalence in the general population.

## Molecular pathways

Besides serrated polyps, conventional adenomas are also common findings in HPS. Due to this heterogeneity, it is unknown which polyps eventually lead to CRC in HPS and thus are clinically relevant. We aimed to analyze which polyps lead to CRC in HPS patients by performing combined immunohistological and molecular analyses. We showed that different molecular pathways are operational in HPS but that the serrated CRC pathway predominates.

## Endoscopic imaging

Considering the presumed increased risk of malignant progression of polyps in HPS, detection and removal of polyps seems necessary to prevent CRC development in these patients. Besides following general quality guidelines of colonoscopy, novel advanced endoscopic techniques, such as narrow-band imaging (NBI) may improve the detection of polyps in HPS. In addition to improved detection of polyps in HPS, accurate differentiation of HPs and SSAs, which appear endoscopically very similar, may aid the endoscopist in only removing SSAs and leaving HPs, which display comparatively lower levels of genetic mutations, in situ. We evaluated the value of NBI and autofluorescence imaging for the detection and differentiation of polyps in HPS.

